# Vasculitic Neuropathy With Iron Deficiency-Related Chronic Inflammation Followed by Elevated Serum Vascular Endothelial Growth Factor: A POEMS (Polyneuropathy, Organomegaly, Endocrinopathy, Monoclonal Gammopathy, and Skin Changes) Syndrome Mimic

**DOI:** 10.7759/cureus.85384

**Published:** 2025-06-05

**Authors:** Keisuke Tachiyama, Takamichi Sugimoto, Atsuko Motoda, Takashi Kurashige, Shiro Aoki, Hirofumi Maruyama

**Affiliations:** 1 Department of Clinical Neuroscience and Therapeutics, Hiroshima University, Hiroshima, JPN; 2 Department of Neurology, Saiseikai Hiroshima Hospital, Hiroshima, JPN; 3 Department of Neurology, National Hospital Organization Kure Medical Center and Chugoku Cancer Center, Kure, JPN

**Keywords:** ascites, iron-deficiency, microscopic polyangiitis, nerve biopsy, vascular endothelial growth factor, vasculitic neuropathy

## Abstract

The causes of peripheral neuropathy are diverse and include numerous systemic diseases. Polyneuropathy, organomegaly, endocrinopathy, monoclonal gammopathy, and skin changes (POEMS) syndrome is a pertinent condition and is characterized by elevated serum vascular endothelial growth factor (VEGF). However, several other diseases with elevated VEGF have been reported besides POEMS syndrome. We report a case of vasculitic neuropathy with iron deficiency-related chronic inflammation, refractory ascites, and high VEGF levels, which was difficult to differentiate from POEMS syndrome. A 66-year-old female presented with fever, lower leg edema, and intractable ascites. She had also experienced tingling sensations and muscle weakness in the right hand and lower limbs in the course of her illness. Neurological findings revealed distal muscle weakness, abnormal sensations, and loss of lower limb tendon reflexes and vibrations. Blood tests revealed anemia with iron deficiency, low unsaturated iron-binding capacity, and high ferritin. The serum VEGF level was markedly elevated, and myeloperoxidase anti-neutrophil cytoplasmic antibody, M protein, and anti-Sjögren’s syndrome-B antibodies were positive. Nerve conduction studies showed axonal impairment, predominantly in the lower limbs, whereas nerve ultrasonography revealed extensive nerve thickening in the extremities. A definitive diagnosis was not reached despite these investigations. The lower limb nerve and muscle biopsies led to the diagnosis of vasculitic neuropathy. In this case, a nerve biopsy was important for a definitive diagnosis. It should be noted that serum VEGF may be elevated not only in POEMS syndrome but also in other disorders such as vasculitic neuropathy and iron deficiency-related chronic inflammation.

## Introduction

Peripheral neuropathy is routinely encountered in neurological practice, and it can have a wide range of causes. Among these, many diseases also affect multiple organs, with polyneuropathy, organomegaly, endocrinopathy, monoclonal gammopathy, and skin changes (POEMS) syndrome being a key example. POEMS syndrome is caused by abnormalities in plasma cells within the bone marrow and manifests various symptoms. The common symptoms include progressive peripheral neuropathy, organ enlargement (such as the liver or spleen), hormonal imbalances, extravascular fluid retention, abnormal plasma proteins, and characteristic skin changes like hyperpigmentation or thickening [1]. Patients with POEMS syndrome show elevated serum levels of vascular endothelial growth factor (VEGF), which is a crucial diagnostic finding [2]. However, in recent years, elevated serum VEGF levels have been reported not only in POEMS syndrome but also in other neuropathies, often making it challenging to differentiate peripheral nerve disorders [3,4]. We report a case of polyneuropathy, refractory ascites, and high serum VEGF levels, which prompted an initial suspicion of POEMS syndrome; however, the patient was diagnosed with vasculitic neuropathy related to iron deficiency following nerve biopsy findings.

## Case presentation

The patient was a 66-year-old female who had developed swelling in the lower limbs and a fever two months before admission to the hospital. She had been initially admitted to another hospital, where a CT scan had shown marked ascites. She had been treated with antibiotics and intravenous albumin following the diagnosis of urinary tract infection and ascites due to liver cirrhosis. However, the patient had shown no improvement and experienced tingling sensations and muscle weakness in the right hand and lower limbs two weeks before being transferred to our hospital for a detailed examination.　

The patient was 153 cm tall and weighed 68.5 kg, with a BMI of 29.3 kg/m^2^. The vital signs were within normal limits, and there were no color changes in the skin, as seen in POEMS syndrome; however, there was pitting edema from both knees to the periphery. Neurological findings showed peripheral muscle weakness in the extremities but no clear laterality. Sensory examination revealed tingling sensations extending from the right palm to 1-4 fingers and distally from both knees. Lower limb vibratory sensation was markedly decreased, and lower limb tendon reflexes were absent. Romberg’s sign was also observed. The patient was unable to walk because of sensory ataxia. The laboratory findings are summarized in Table 1.

**Table 1:** Laboratory findings on admission Anti-SS-B: anti-Sjögren's syndrome B; MPO-ANCA: myeloperoxidase antineutrophil cytoplasmic antibody; UIBC: unsaturated iron-binding capacity; VEGF: vascular endothelial growth factor

Investigation	Patient value	Reference range
White blood cell (/μL)	13250	3300-8600
Red blood cell (/μL)	2.93 × 10^6^	3.86-4.92 × 10^6^
Hemoglobin (g/dL)	9.5	11.6-14.8
Platelet (/μL)	16.0 × 10^4^	15.8-34.8 × 10^4^
Aspartate aminotransferase (U/L)	61	13-30
Alanine aminotransferase (U/L)	36	7-23
Blood urea nitrogen (mg/dL)	12.9	8-20
Creatinine (mg/dL)	0.56	0.46-0.79
Sodium (mmol/L)	133	138-145
Potassium (mmol/L)	3.1	3.6-4.8
Iron (µg/dL)	21	40-188
UIBC (µg/dL)	100	130-370
Ferritin (ng/mL)	624.4	11.8-128.9
C-reactive protein (mg/dL)	6.7	0-0.14
Erythrocyte sedimentation rate (mm/h)	70	3-15
Immunoglobulin G (mg/dL)	2204	861-1747
Immunoglobulin M (mg/dL)	111	50-269
Immunoglobulin A (mg/dL)	657	93-393
Albumin (%)	44.3	60.2-71.4
Alpha 1 globulin (%)	3.7	1.9-3.2
Alpha 2 globulin (%)	8.0	5.8-9.6
Beta globulin (%)	7.6	7.0-10.5
Gamma globulin (%)	36.4	10.6-20.5
Anti-SS-B antibodies (U/mL)	26.8	0-9.9
MPO-ANCA (U/mL)	15.5	0-3.4
Serum VEGF (pg/mL)	2119	143.1-658.8

Complete blood count revealed a mild increase in white blood cell count, but no increase in the eosinophil count. The red blood cell (RBC) count and hemoglobin (Hb) levels were decreased. The platelet count was within the normal limit. A mild elevation in liver enzymes, hyponatremia, and hypokalaemia was observed in the biochemical analysis; however, hepatitis B and C virus antibodies were negative. Blood urea nitrogen and creatinine were normal. The serum iron level and unsaturated iron-binding capacity (UIBC) were low. Serum ferritin levels were high. C-reactive protein and erythrocyte sedimentation rate were elevated.

Abnormal laboratory findings that could indicate peripheral neuropathy included polyclonal gammopathy of immunoglobulin, a small amount of immunoglobulin G kappa-type M protein, positive anti-Sjögren’s syndrome (SS)-B antibodies, and positive myeloperoxidase antineutrophil cytoplasmic antibody (MPO-ANCA). We suspected POEMS syndrome owing to the presence of leg edema, ascites, and peripheral neuropathy. The serum VEGF levels were also significantly elevated. The urinalysis showed no abnormalities in both qualitative and sedimentation components. Cerebrospinal fluid (CSF) examination showed no elevation of cell count or protein. Spinal fluid cytology was negative. The bone marrow puncture was performed from the left iliac bone, but no plasma cell proliferation was observed. CT showed marked ascites and liver atrophy; however, no obvious enlargement of organs or presence of tumors was found. There were no findings suggestive of pneumonia.

**Figure 1:**
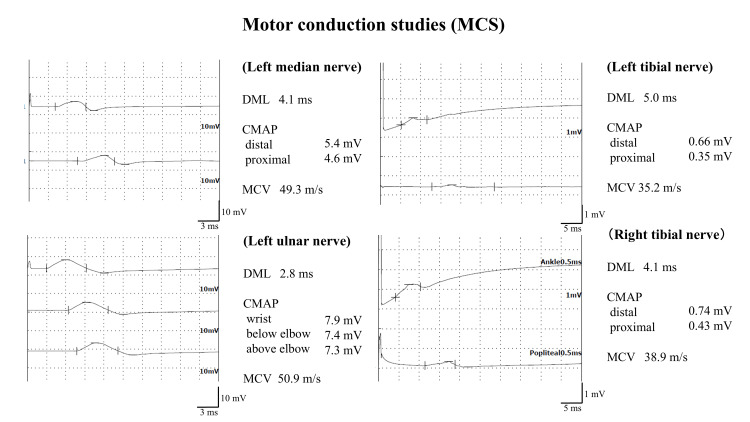
Motor conduction studies of the left upper and both lower limbs CMAP: compound muscle action potential; DML: distal motor latency; MCV: motor conduction velocity

Nerve conduction studies revealed a significant reduction in the compound muscle action potential (CMAP) amplitude of motor nerve in both tibial nerves and a mild reduction in CMAP amplitude of motor nerves in the left median and ulnar nerves (Figure 1). 

**Figure 2:**
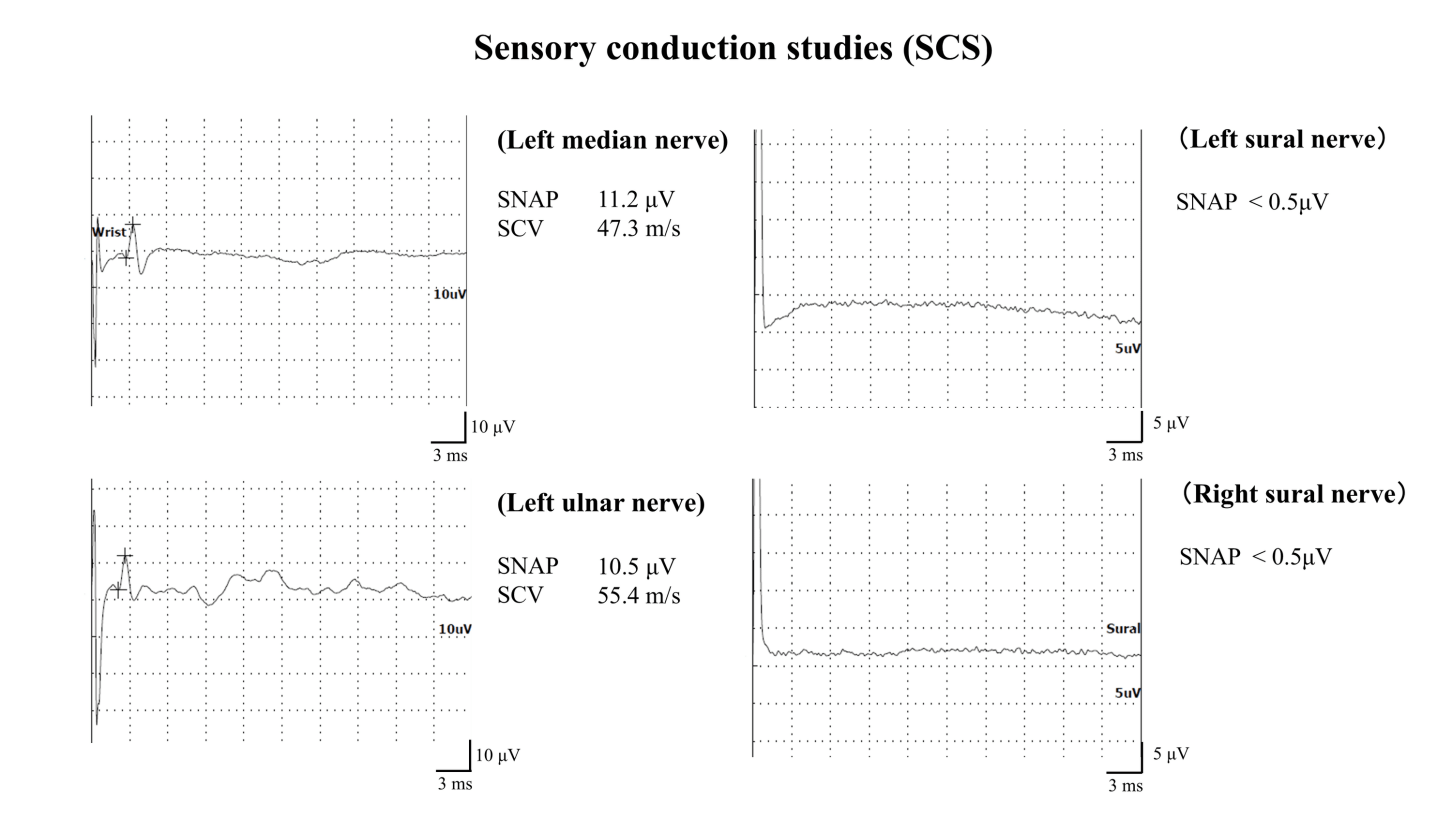
Sensory conduction studies of the left upper and both lower limbs SCV: sensory conduction velocity; SNAP: sensory nerve action potentials

The sensory conduction studies presented no sensory nerve action potentials in the bilateral sural nerve, though there were no abnormalities in the left median and ulnar nerves (Figure 2).

**Figure 3:**
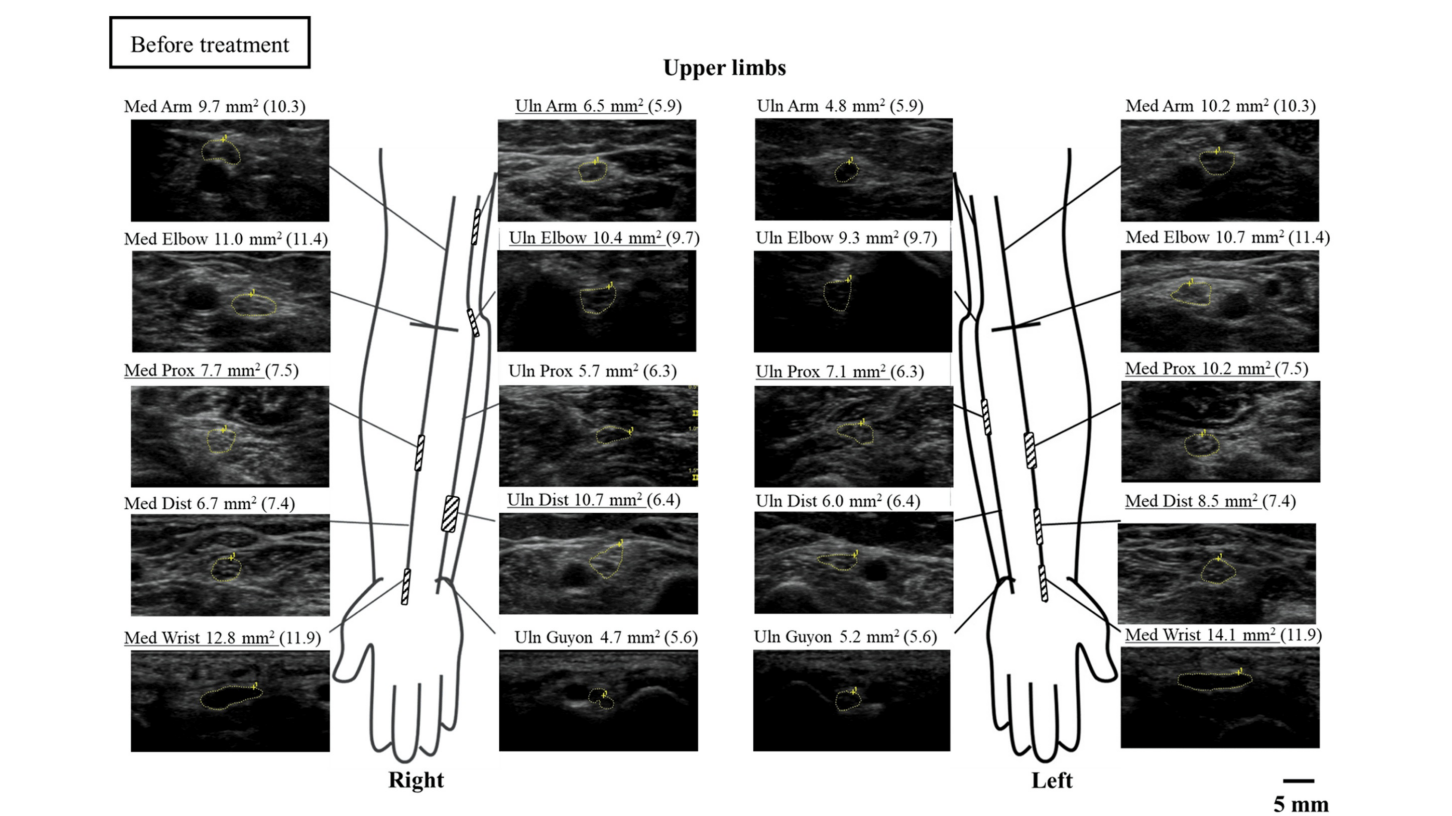
Nerve ultrasound findings in the upper extremities before treatment The median and ulnar nerves are measured. The values in parentheses indicate the respective cutoff values for the CSA. The cutoff values and measurement sites for each nerve are based on the studies by Sugimoto et al. [5]. The measurement sites of CSA are illustrated. Underlined CSA values indicate measurements that exceed the respective cutoff values Dist: distal; CSA: cross-sectional area; Med: median nerve; Prox: proximal; Uln: ulnar nerve

In the nerve ultrasonographic examination, patchy enlargements of the cross-sectional area (CSA) were observed in the bilateral median and ulnar nerves before treatment (Figure 3).

**Figure 4:**
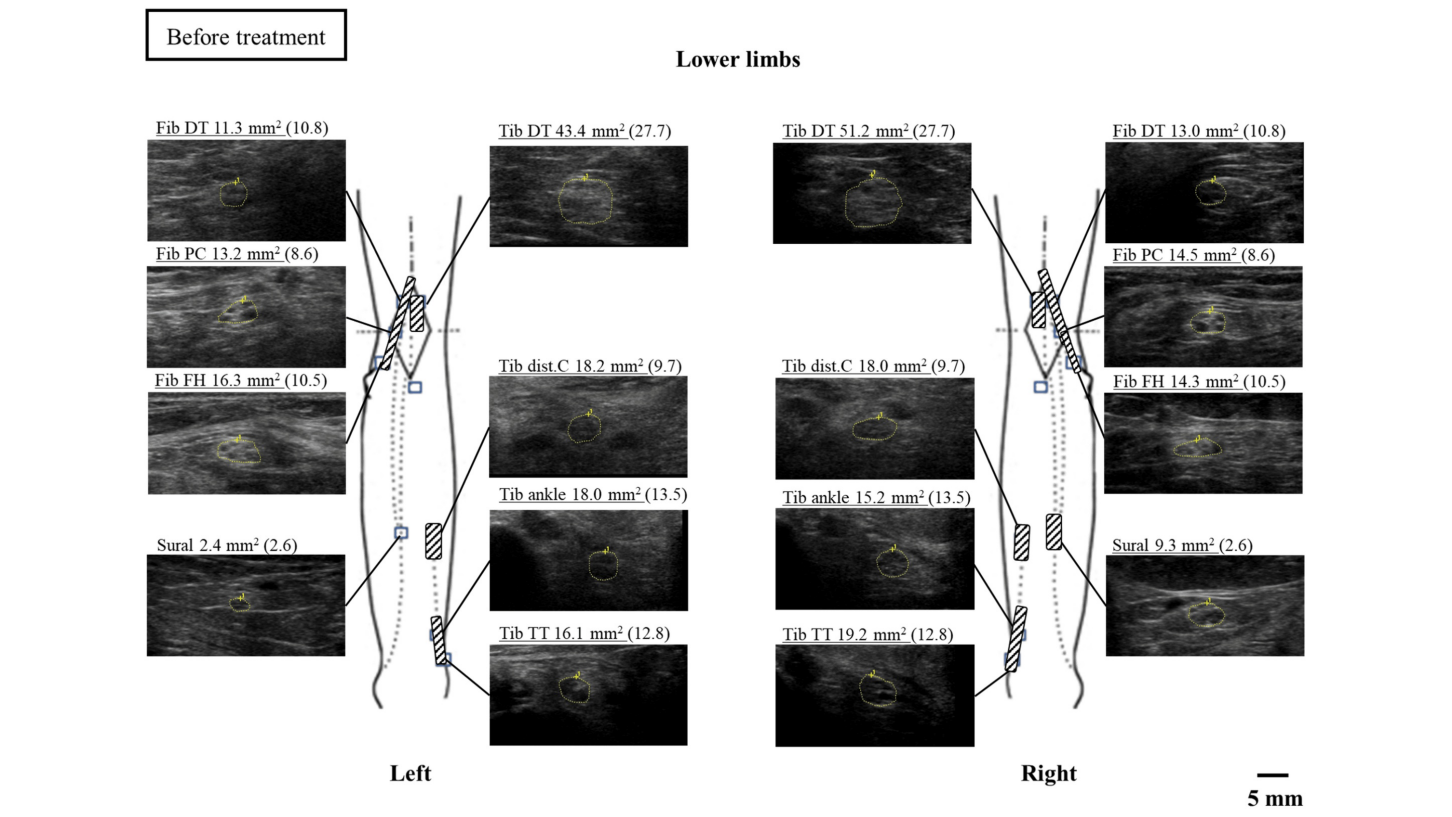
Nerve ultrasound findings in the lower extremities before treatment The tibial, fibular, and sural nerves are measured. The cutoff values and measurement sites for each nerve are based on the studies by Kuga et al. [6]. The measurement sites of CSA are illustrated. Underlined CSA values indicate measurements that exceed the respective cutoff values CSA: cross-sectional area; dist C: distal calf; DT: distal thigh; FH: fibular head; Fib: fibular nerve; PC: popliteal crease; Tib: tibial nerve; TT: tarsal tunnel

Enlargement of the CSA was also observed in the bilateral tibial and peroneal nerves at all measurement sites and the right sural nerve (Figure 4).

**Figure 5:**
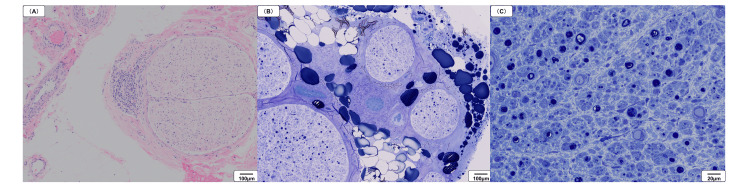
Pathological findings of the left sural nerve (A) Perivascular cuffing due to inflammatory cell infiltration of the epineurium (hematoxylin and eosin staining; ×100). (B) Reduction in myelinated nerve density (toluidine blue stain; ×100). (C) Myelin ovoids (toluidine blue stain; ×400)

The cause of the polyneuropathy could not be definitively identified despite a detailed examination; therefore, a sural nerve biopsy was performed on the left side, where edema was less pronounced, for a definitive diagnosis (Figure 5). Perivascular cuffing due to inflammatory cell infiltration of the epineurium was observed (Figure 5A). The myelinated nerve density was severely reduced in all nerve bundles, some of which were heterogeneously reduced (Figure 5B). In addition, many of the remaining myelinated nerves formed myelin ovoids (Figure 5C). There were no demyelinating findings suggestive of POEMS syndrome. Based on these findings, we finally established a diagnosis of vasculitic neuropathy.

**Figure 6:**
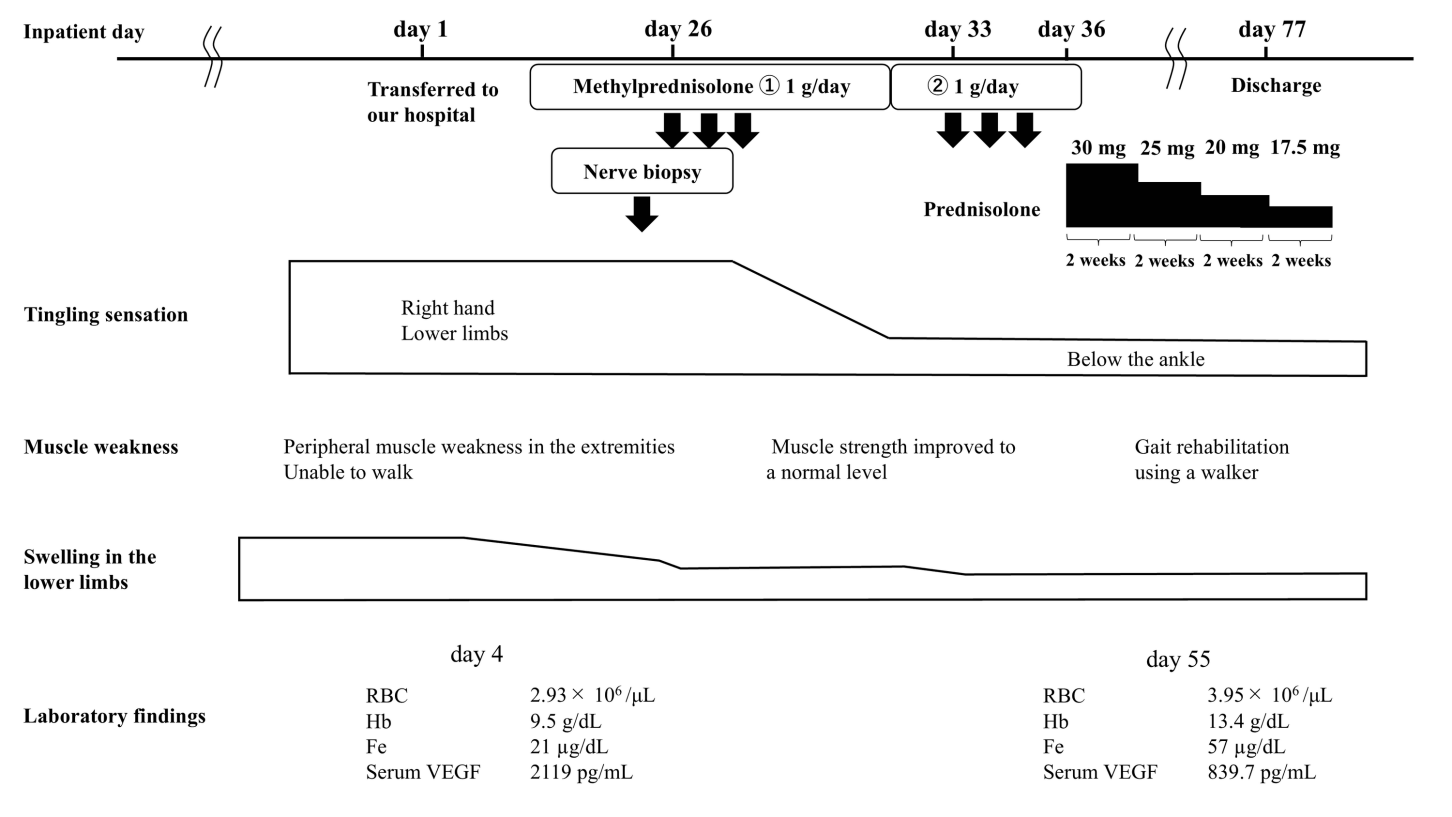
Clinical course and treatment Fe: serum iron; Hb: hemoglobin; RBC: red blood cell; VEGF: vascular endothelial growth factor

A three-day course of high-dose intravenous methylprednisolone (1 g/day) treatment was started on the day after the nerve biopsy (Figure 6).

**Figure 7:**
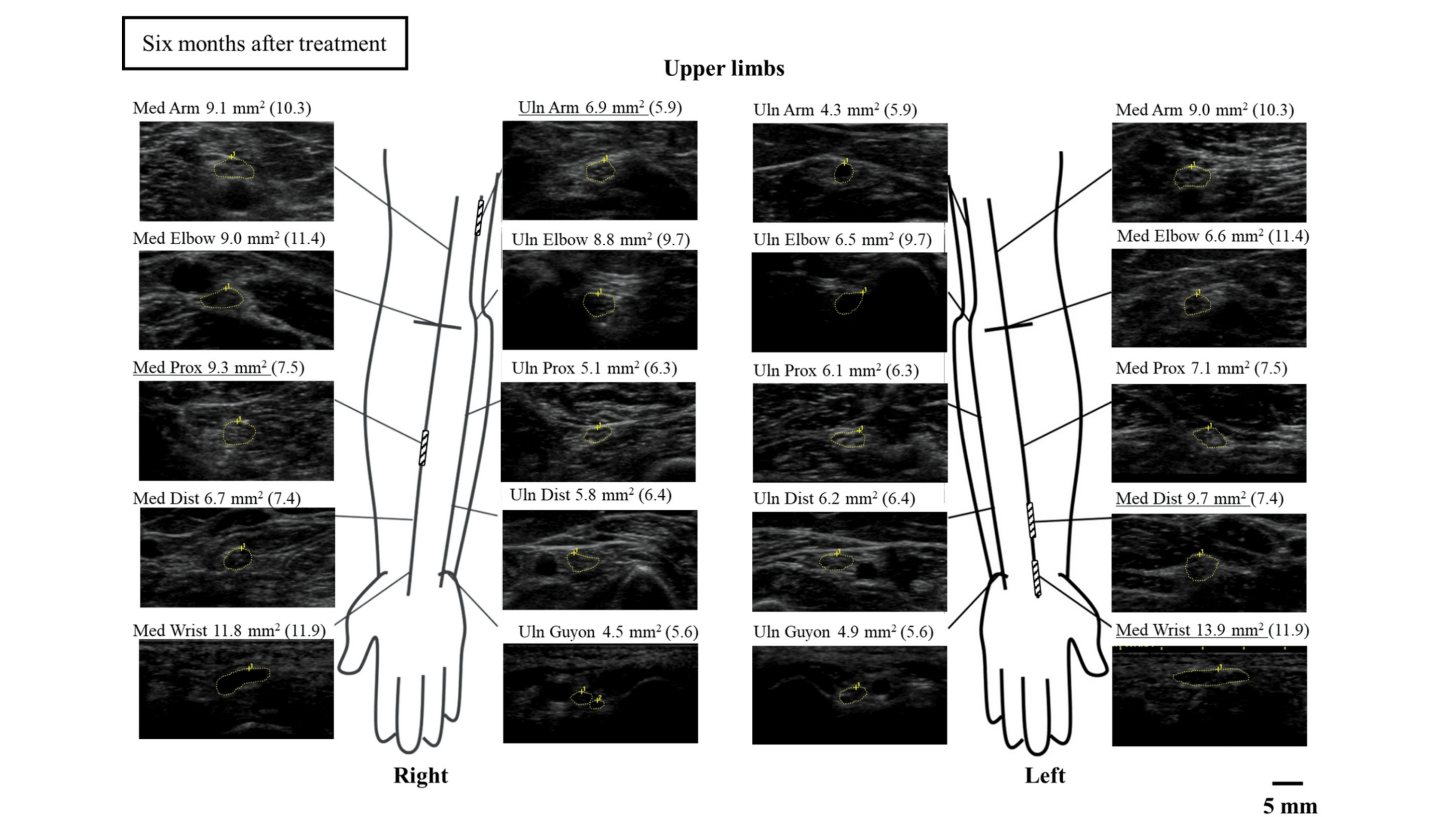
Nerve ultrasound findings in the upper extremities six months after treatment Med: median nerve; Uln: ulnar nerve

**Figure 8:**
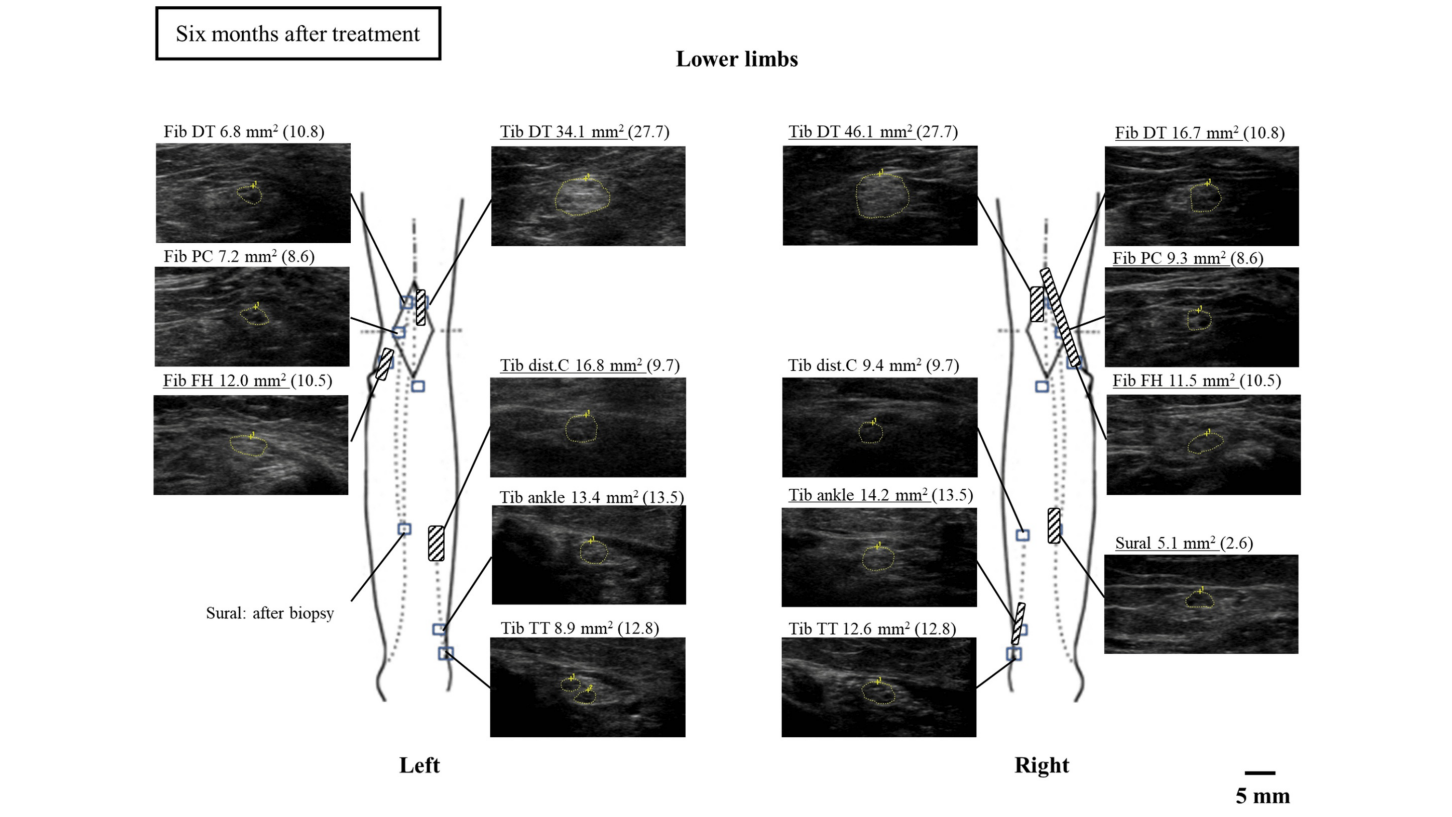
Nerve ultrasound findings in the lower extremities six months after treatment dist C: distal calf; DT: distal thigh; FH: fibular head; Fib: fibular nerve; PC: popliteal crease; Tib: tibial nerve; TT: tarsal tunnel

The treatment included a total of two courses. After one course of intravenous methylprednisolone treatment, the tingling sensation was reduced to below the ankle, and muscle strength improved to a normal level. After the second course of treatment, the patient underwent gait rehabilitation using a walker. Re-evaluation of the neuro-ultrasound six months after treatment showed that the nerve enlargement along the upper and lower limbs had partially decreased (Figures 7, 8).

Serum VEGF levels were re-evaluated six months after treatment and showed a marked decrease to 839.7 pg/mL. There was no history of excessive alcohol consumption and no significant abnormalities in the blood tests; although a liver biopsy was considered, it could not be performed due to the large amount of ascites, and the cause remained unclear. A significant improvement was observed with intravenous albumin, intensified diuretics, and nutritional therapy, including branched-chain amino acid preparations. The anemia and iron deficiency improved with treatment for vasculitis and without oral iron preparation. (RBC: 3.95 × 10^6^ /μL, Hb: 13.4 g/dL, Fe: 57 μg/dL).

## Discussion

There are many causes of neuropathy, which include vasculitic neuropathy and POEMS syndrome. Vasculitic neuropathy is an ischemic disease caused by the inflammation of small arteries and arterioles. The typical clinical presentation of vasculitic neuropathy includes sensory symptoms that are more severe than motor symptoms, exhibiting asymmetry and severe pain in the distal parts of the limbs. These symptoms progress rapidly and commonly demonstrate the clinical features of multifocal mononeuropathy [7]. In our case, the abnormal sensation observed exclusively in the right upper limb may have been indicative of the asymmetrical symptom characteristic of vasculitis. However, our patient did not present with any pain.

POEMS syndrome presents with various symptoms in addition to peripheral neuropathy, including edema, ascites, organ enlargement, and skin symptoms [1]. Our patient had lower leg edema and ascites; therefore, this case met two major criteria for POEMS syndrome (polyneuropathy and elevated serum VEGF) and one minor criterion (edema), strongly suggesting a diagnosis of POEMS syndrome [8]. Blood tests showed elevated VEGF levels and MPO-ANCA positivity as well as a small amount of IgG kappa-type M protein and positive anti-SS-B antibodies, contributing to the difficulty in confirming the diagnosis. The patient was also found to have liver cirrhosis, which is generally found to present with hyper-γ-globulinemia [9]. Hyper-globulinemia was thought to have resulted in the detection of a small amount of IgG kappa-type M protein.

Regarding the positive SS-B antibody, the Schirmer test and lip biopsy were also performed; however, no abnormal findings were observed, and it was concluded that the patient did not have SS-related neuropathy. Peripheral neuropathy was identified as a major symptom, while pulmonary and renal involvement was not observed. According to the American College of Rheumatology and the European Alliance of Associations for Rheumatology (ACR/EULAR) classification criteria, even if there are no characteristic lung or kidney lesions of microscopic polyangiitis (MPA), if MPO-ANCA is positive, the case can be classified as MPA [10]. However, in the definitive diagnostic criteria for MPA used in Japan, the presence of pulmonary or renal lesions is crucial for diagnosis. Our patient did not present with systemic symptoms (such as fever and weight loss) or pulmonary and renal involvement that are frequently observed in MPA, and only showed findings of multiple mononeuropathies. Therefore, in addition to POEMS syndrome, MPO-ANCA-associated vasculitic neuropathy was also considered as a differential diagnosis.

Nerve conduction studies generally show asymmetric, non-length-dependent axonal motor-sensory neuropathy in vasculitic neuropathies [11]. In contrast, POEMS syndrome is length-dependent, meaning that longer nerves are more prone to damage and intense axonal motor sensory neuropathy in the lower limbs [12]. In our case, axonal impairment was pronounced in the lower limbs without significant asymmetry. The differentiation of vasculitic neuropathy from POEMS syndrome based on clinical, biochemical, and electrophysiological findings was found to be challenging. Therefore, a nerve biopsy was essential for a definitive diagnosis. The neuropathological findings showed evidence of vasculitis with infiltration of inflammatory cells into the vessel walls and surrounding areas. Additionally, a heterogeneous reduction in nerve fiber density and the distribution of myelin ovoids suggested vasculitic neuropathy [13]. In contrast, POEMS syndrome is characterized not only by axonal degeneration but also by demyelination, particularly in the proximal segments of the peripheral nerves [14]. In this case, nerve fiber teasing technique was not performed, but no findings suggestive of demyelination were observed.

In this case, a nerve ultrasonographic examination was performed before treatment and six months after, revealing a partial improvement in nerve enlargement. There have been a few reports of improvement in nerve enlargement before and after treatment for vasculitic neuropathy [15]. This suggests that nerve ultrasonographic examination, in addition to clinical symptoms and electrophysiological findings, is a useful tool for post-treatment evaluation.

VEGF is a glycoprotein cytokine involved in angiogenesis. In general, high serum VEGF levels are characteristic of POEMS syndrome [2]. The elevated serum VEGF levels promote angiogenesis and increase vascular permeability. Sakai et al. have reported that plasma VEGF levels were elevated in vasculitic neuropathy, suggesting the potential utility of plasma VEGF as a diagnostic and therapeutic marker [16]. Unlike plasma, serum is obtained by coagulating blood and contains VEGF released from platelets during coagulation. In POEMS syndrome, platelets aggregate on vascular endothelial cells, which secrete platelet-derived VEGF (VEGF-A), resulting in markedly high serum VEGF levels [17]. To date, an elevated “serum” VEGF level in vasculitic neuropathy has not been reported. However, Ueno et al. have reported that in Kawasaki disease, a pediatric vasculitis, there is a significant increase in serum VEGF, particularly VEGF-A, which is released during platelet aggregation at sites of vasculitis inflammation [18]. Therefore, serum VEGF levels, particularly VEGF-A, might also increase through a similar mechanism in vasculitic neuropathy.

Several reports have documented increased serum VEGF levels in other neuropathies, such as chronic inflammatory demyelinating polyneuropathy and myelin-associated glycoprotein-positive neuropathy [3,4]. In addition to neurological diseases, other causes of elevated serum VEGF levels have been reported, including tumors, iron-deficiency anemia, untreated hypoxemic diseases (obstructive sleep apnea-hypopnea syndrome and chronic obstructive pulmonary disease) [4], and chronic inflammatory diseases such as rheumatoid arthritis (RA) [19]. Diseases associated with elevated serum VEGF levels are diverse, and various factors can cause elevated serum VEGF in addition to plasma cell production, one of which is hypoxia.

The oxygen-carrying capacity of the tissues is reduced in iron deficiency, leading to tissue hypoxia, which activates hypoxia-inducible factor-1 alpha, a transcription factor that stimulates VEGF secretion [20]. Pihan et al. have reported that serum VEGF is significantly elevated in patients with anemia and low serum iron [789 pg/mL (reference range: 320-2616 pg/mL)] [4]. Another factor is the involvement of inflammatory cytokines. In patients with RA, synovial changes include neovascularization, and a significant increase in VEGF levels has been reported to correlate with disease activity [19]. A study using synovial fibroblasts derived from patients with RA reported that IL-6, an inflammatory cytokine, may stimulate the secretion of serum VEGF [21].

In our patient, the low serum iron and UIBC and high serum ferritin suggest that the anemia was associated with vasculitis as a chronic inflammatory disease. We considered that vasculitis and the iron deficiency resulting from this inflammation may also lead to the elevation of serum VEGF.

## Conclusions

We discussed a case of vasculitic neuropathy associated with iron deficiency, which was challenging to differentiate from POEMS syndrome. Characteristic symptoms and diagnostic findings for each neurological disorder are not always present, making a definitive diagnosis through a nerve biopsy crucial. Moreover, nerve biopsy plays an important role in determining the appropriate course of treatment. Although elevated serum VEGF is generally considered a characteristic finding of POEMS syndrome, it may also be elevated in diseases other than POEMS syndrome due to the various mechanisms that contribute to serum VEGF elevation. Therefore, this should be taken into account when diagnosing peripheral neuropathy.
